# Common Sensitive Diagnostic and Prognostic Markers in Hepatocellular Carcinoma and Their Clinical Significance: A Review

**DOI:** 10.7759/cureus.23952

**Published:** 2022-04-08

**Authors:** Basim Saleh Samman, Albadr Hussein, Razan Saleh Samman, Abdulaziz Saud Alharbi

**Affiliations:** 1 Internal Medicine Department, College of Medicine, Taibah University, Al-Madinah Al-Munawwarah, SAU; 2 Medicine, King Fahad General Hospital, Al-Madinah Al-Munawwarah, SAU; 3 General Surgery Department, College of Medicine, Taibah University, Al-Madinah Al-Munawwarah, SAU

**Keywords:** liver cancer biomarkers, des-gamma carboxyprothrombin, alpha-fetoprotein, hepatocellular carcinoma, liver cancer

## Abstract

Liver cancer is one of the most serious cancers that lead to death around the world. In Saudi Arabia, it represents 4.1% of all diagnosed cancers in 2020. The total survival rate for all stages of liver cancer is 15% at five years after diagnosis, and this can be affected by the available therapy. Hepatocellular carcinoma (HCC) is known to be the most prevalent primary malignant tumor of the liver, and several studies have been conducted to improve the management approach in the early and late stages. Biomarkers are a useful tool for early diagnosis, disease progression, prognosis, and targeted therapy for patients with liver cancer. The most important biomarker that has been studied is alpha-fetoprotein (AFP), which is elevated in 70% of patients with liver cancer. Also, des-gamma-carboxyprothrombin (DCP) is a more specific biomarker in HCC compared to AFP as it is elevated in HCC, which can be elevated in several conditions that cause active hepatitis. In addition, squamous cell carcinoma antigen (SCCA) and Golgi protein 73 (GP73) have high sensitivity compared to AFP and DCP but poor specificity. All of these markers are useful in the diagnosis, management, and prognosis prediction of liver cancer, especially the combination of AFP and DCP.

## Introduction and background

Liver cancer is one of the most serious cancers that lead to death worldwide [1]. In Saudi Arabia, it represents 4.1% of all diagnosed cancers in 2020 [2]. There are two types of liver cancer: primary liver cancers arise from the liver, of which hepatocellular carcinoma (HCC) is considered as the most common primary liver cancer in adults [3,4]. Secondary liver cancers are usually metastases from other sites and are more common than primary types. The most common primary sites are the stomach, pancreas, breasts, colon, bladder, and lungs [3-5]. Excessive alcohol intake is the major risk factor for primary liver disease along with cirrhosis, obesity, diabetes, and hepatitis B viral (HBV) infection or hepatitis C viral (HCV) infection [3,6,7].

Despite advanced studies, the exact cause of liver cancer is still unknown [6]. The common symptom of liver cancer is pain, particularly in the upper right hypochondrium. Also, unexplained weight loss and palpable mass in the subcostal region in large cancers [3,4]. Tumor-node-metastases (TNM) staging of liver cancer is usually used in the management and prediction of prognosis [8]. The diagnosis of liver cancer is mainly dependent on imaging modalities such as CT or MRI and taking a biopsy. In addition, serum tests can be used, such as alpha-fetoprotein (AFP), which might be raised in 70% of patients with liver cancer [5,8]. The therapeutic approach depends on the stage of the tumor in addition to the age and functional status of the patient [5,9]. The prognosis for liver cancer relies on the stage of presentation, liver function, and the presence or absence of co-morbidities [5]. According to American Cancer Society guidelines, the total survival rate for all stages of liver cancer is 15% at five years after diagnosis. Also, the survival rate can be affected by the available treatments. For example, after a liver transplant, the five-year survival rate can be as high as 70% [5]. Liver cancer can be prevented by limiting alcohol intake, maintaining a healthy weight, and getting vaccines against hepatitis B [6,9].

Multiple biomarkers may play a role in liver cancer diagnosis and outcome [10-15]. The biomarkers that have been evaluated in HCC are fucosyltransferase 1 (FUT1), beta-1,3-galactosyltransferase 5 (B3GALT5) [11], CUG triplet repeat-RNA binding protein 1 (CUGBP1) [12], death-associated protein kinase 1 (DAPK1) mRNA [12], transforming growth factor-beta 1 (TGF-β1) [13], squamous cell carcinoma antigen (SCCA), serum anti-p53, hepatocyte growth factor [14], alpha-fetoprotein (AFP) [13-15], des-gamma-carboxyprothrombin (DCP) [14,15], and Golgi protein 73 (GP73) [13,14]. Our objectives in this article are to review HCC, the most sensitive diagnostic and prognostic markers of HCC, and its clinical significance.

## Review

Hepatocellular carcinoma

Hepatocellular carcinoma (HCC) is the most prevalent primary malignant tumor of the liver and the sixth most common cancer worldwide [3]. Also, HCC is the second leading cause of cancer-related death [14]. HCC is more prevalent in males than in females, with a ratio of 4:1, and the peak age of presentation is in the fifth to sixth decades of life. However, it can be present earlier in areas with high prevalence regions of HBV and HCV infections. Furthermore, cirrhosis increases the risk of HCC in 70-80% of cases [3]. Despite the advanced studies, therapy options for advanced HCC are still ineffective, leading to a poor prognosis and only about one-third of patients are eligible for therapy [14]. Improving our knowledge about markers may eventually result in an early diagnosis and preferable therapeutic strategies for this deadly illness [16,17].

Clinical pictures

The patients remain asymptomatic in the early stages. However, when the disease manifests, they may present with [9]: loss of weight without obvious causes or dieting, right hypochondriac pain close to the right shoulder or in the back, jaundice, decreased appetite for eating, and general tiredness. 

Etiological pathogenesis 

The most significant etiologies of HCC are hepatitis B (HBV) and hepatitis C viral (HCV) infection, and they are correlated with cirrhosis [3]. Also, excess alcohol consumption, diabetes, and obesity are among the risk factors for the development of HCC [3]. All of these factors contribute to an incessant state of inflammation and fibrogenesis, resulting in fibrosis and cirrhosis, pre-neoplastic states that precede the development of HCC. Therefore, patients with chronic hepatitis have a significant risk of developing liver cirrhosis and, ultimately, HCC [16,17].

Relation to HBV infection

The genesis of HCC is related to chronic infection with HBV. HBsAg positivity is greater in HCC patients. In contrast, in developing countries, HBV is as yet the superior etiologic agent in the pathogenesis of HCC. In Taiwan, HBsAg-positive carriers have a more than 200 times higher risk of HCC development than HBsAg-negative patients, especially when the infection is acquired early in life. In Asian and African patients, 95% of cases of HCC have anti-HBc [3].

Relation to HCV infection

Chronic HCV infection has emerged as a major agent in the etiology of HCC, mostly after more than 30 years of infection. In developed countries, there is a rising incidence of HCC due to rising HCV infections. Patients having anti-HBc and anti-HCV antibodies jointly have a three times higher risk of developing HCC than those with either antibody alone. HCV infection subsequent to a prolonged interval leads to cirrhosis more predominating prior to the promotion of HCC. However, in HCC after HBV infection, half the status has cirrhosis and the residue have chronic hepatitis.

Relation to cirrhosis

Cirrhosis of all etiologic forms is more usually correlated with HCC, but the most frequent correlation is with macronodular post-necrotic cirrhosis. The mechanism of advancement to HCC showed to be chronic regenerative activity in cirrhosis. Liver cell dysplasia has been identified by cellular magnification, nuclear hyperchromatism, and multinucleate cells, and is found in 60% of cirrhotic livers with HCC and only 10% of non-cirrhotic livers [3]. 

Staging

For the staging of liver cancer, the TNM system helps in the management and prediction of prognosis. T means tumor, N means diffused to lymph nodes, and M means metastasis to other sites in the body. T1 indicates a single tumor equal to 2 cm or less, and T2 indicates single or multiple tumors more than 2 cm but less than 5 cm. T3 indicates more than one tumor, with at least one tumor greater than 5 cm. T4 indicates at least one tumor of any size that is mature in the portal or hepatic vein, and N1 means that the tumor has diffused to close lymph nodes. Lastly, M1 means the tumor has diffused to distant organs [8]. 

Management 

The management of the HCC is based on the stage of the disease as well as age, general health, and personal predilection.

Surgery

Aimed to remove the tumor and an edge of well tissue, this besetment might be a choice for a person with early extent HCC who have a good liver function.

Liver Transplant Surgery

Aiming to remove the whole liver and substitute it with a liver from a granter might be a choice in a healthy person whose HCC has not diffused away from the liver.

Radiation Therapy

This choice depends on the high power of sources like Roentgen rays and protons to break down tumor cells and shrink cancer. Also, it might be recommended if surgery is not a choice [9].

Prevention

Hepatic cancers may be prevented by decreasing exposure to recognized risk factors, but there are still no reliable preventive measures.

Eschew and Handling Hepatitis Infections

The most important hazard agent for liver cancer in the worldwide is chronic infection with HBV and HCV. However, exposure to these viruses can be prevented via prohibiting the participation of needles, taking vaccines for HBV that decrease the hazard of hepatitis, avoiding unprotected sex, and screening of blood transitions [8].

Limiting Tobacco and Alcohol Use

Consuming alcohol in a moderate amount might help to restrain HCC. Furthermore, smoking cessation may decrease the development of HCC [8].

Maintain a Healthy Weight

Averting obesity may be useful to safeguard versus HCC. An obese person has more chance to develop diabetes and fatty liver illness; each other has been correlated to HCC [8]. 

Review of biomarkers

Biomarkers of hepatocellular carcinoma and their clinical applications and the review of biomarkers are given in Table 1.

**Table 1:** Biomarkers of hepatocellular carcinoma and their clinical applications. AFP: alpha-fetoprotein; DCP: des-gamma-carboxyprothrombin; SCCA: squamous cell carcinoma antigen; GP73: Golgi protein 73.

Application	Specificity (%)	Sensitivity (%)	Markers
Early diagnosis	80-94%	41-65%	AFP
Prognosis	81-98%	48-62%	DCP
Early diagnosis	49%	84%	SCCA
Diagnosis	69%	75%	GP73

Alpha-fetoprotein (AFP)

Physiologically, it is manufactured during the early extent of fetal liver growth via the endodermal cells of the visceral yolk sac. The AFP is expressed via hepatocytes and endodermal cells from the yolk sac reduction after childbirth. The increase of AFP can happen through hepatocarcinogenesis, hepatocyte regeneration, and fetal carcinomas [13,18]. Thus far, the biological role of AFP is still unknown.

Analysis of new studies has specified that AFP testing is less sufficient in specificity and sensitivity for efficient surveillance. The level of AFP is within the normal average in up to 40% of patients with HCC, especially in the early period of the disease (low sensitivity). High AFP might be expressed in patients with aggravation of chronic hepatitis, cirrhosis, or cholangiocarcinoma (low specificity). Also, some studies have shown that AFP has extraordinarily restricted diagnostic precision in detecting little HCC. The elevation of AFP levels of >500 ng/ml is associated with the tumor bulk; 80% of small HCCs show no elevation of AFP concentration. In addition, the sensitivity of AFP reduction was increased from 25% to 52% while tumor size was >3 cm and <3 cm. Several patients with hepatic inflammation and/or cirrhosis may have an increased AFP without the existence of the tumor. The AFP serum concentration level through the follow-up of patients' post-treatment is a useful screening in conjunction with magnetic resonance imaging or computed tomography. A reduction of AFP grade <10 ng/ml through 30 days post-treatment is considered an appropriate response to treatment. In contrast, the valuation of serum AFP concentration is clinically important when AFP is increased before the therapy [13,14].

Des-gamma-carboxy prothrombin (DCP)

Des-gamma-carboxy prothrombin (DCP) is a defect prothrombin and came from an obtained disorder in the next to translational carboxylation for the prothrombin precursor in HCC cells. DCP measurement of HCC has a sensitivity of 48% to 62% and a specificity of 81% to 98%. DCP is a more specific marker for HCC than AFP because it appears not too high in other liver illnesses. The precision of DCP is the reduction in prolonged obstructive jaundice, intrahepatic cholestasis with vitamin K insufficiency, and ingestion of warfarin. Increased DCP grade in HCC patients is correlated with a bad prognosis. DCP is implicated in tumor angiogenesis and rising genetic expression of angiogenic factors like vascular endothelial growth factor (VEGF), endothelial growth factor-receptor (EGF-R), and matrix metalloproteinase-2 (MMP-2). Also, play a role in contributing to the proliferation and migration of human vascular endothelial cells. DCP-positive patients considerably promote intrahepatic metastasis, capsular infiltration, portal vein invasion, and hepatic vein thrombosis. In a recent article, Hakado et al. propose that the increase of AFP with DCP levels at 24 weeks next to the accomplishment of ribavirin and interferon (IFN) therapy were substantially correlated with the occurrence of HCC irrespective of virological response in Japanese patients with cirrhosis [13,14]. However, the combination of AFP and DCP might be high in sensitivity even in early extent patients [19]. Recently, a meta-analysis specified that DCP had moderate diagnostic precision in HCC. Furthermore, studies with strict design, considerable sample bulk, and multiregional collaboration are necessary for the future [13,14].

Squamous cell carcinoma antigen (SCCA)

Serpins serin protease inhibitors contain squamous cell carcinoma antigen (SCCA) that safeguards tumor cells from apoptosis. SCCA expression, as well as AFP output, may be the sequel of the dedifferentiation predominantly observed in HCC. Propose that the expression of SCCA rises in the precocious grades of HCC formation. In contrast, increased SCCA significance in the tumor was inversely associated with the nodule size. Patients with HCC have a higher SCCA serum grade than cirrhotic patients. There is no obvious association between the SCCA serological standard and SCCA expression in tissues. SCCA might be applied for HCC diagnosis. Also, it appears a sensitivity of 84% and a specificity of 49%. Offered that SCCA is inversely associated with tumor bulk, it is useful for precocious HCC diagnosis and in check of inveterate hepatic illness patients [13,14]. A new meta-analysis specified that SCCA and SCCA IgM show moderate diagnostic precision as new tumor markers of HCC, though the significance of the integration of SCCA/SCCA IgM and AFP needs further research [20]. 

Golgi protein-73

It is a Golgi glycoprotein expressed in epithelial human cells. Physiologically, GP73 is expressed in biliary epithelial cells. However, it is not in hepatocytes. In hepatic illness, GP73 expression is increased in hepatic cells. The GP73 rate is higher in precocious HCC patients than in cirrhotic patients. GP73 is reckoning a potential marker for HCC. In addition, it demonstrates a specificity of 75% and a sensitivity of 69%. There are some isoforms of GP73 associated with various grades of glycosylation [13,14]. In addition, several isoforms are more particular for HCC. Furthermore, additional studies are necessary to emphasize the role of GP73 in HCC diagnosis. In this meta-analysis display that in HCC diagnosis, the precision of GP73 was more than that of AFP, and that GP73 + AFP showed significantly more diagnostic precision than GP73 or AFP alone [20]. Also, we propose that GP73 is a worthy serum marker in patients with HCC, and the serum concentration might also be raised in patients with solid benign liver tumors. Moreover, the GP73 assay is less useful for distinguishing between primary malignant and benign liver tumors [20].

## Conclusions

In conclusion, HCC has a poor prognosis with a survival rate of only 15% five years after diagnosis. Therefore, improving early diagnosis methods is needed. AFP and DCP biomarkers are helpful tools for diagnosis and prognosis in the early stages, but they have poor sensitivity, which cannot role out HCC. DCP is a more specific biomarker in HCC compared to AFP. In addition, SCCA and GP73 markers have a higher sensitivity compared to AFP and DCP but with poor specificity, and need more studies regarding their role in diagnosis and prognosis for HCC. All of these markers are useful tools for diagnosis, prediction of prognosis, and management of HCC.
